# Supplementary material for: RhlR-Regulated Acyl-Homoserine Lactone Quorum Sensing in a Cystic Fibrosis Isolate of Pseudomonas aeruginosa
Source: mBio. 2020 Apr 7;11(2):e00532-20. doi: 10.1128/mBio.00532-20 (PMC7157775; doi:10.1128/mBio.00532-20)
Supplement: FIG S1 [file mBio.00532-20-sf001.docx]

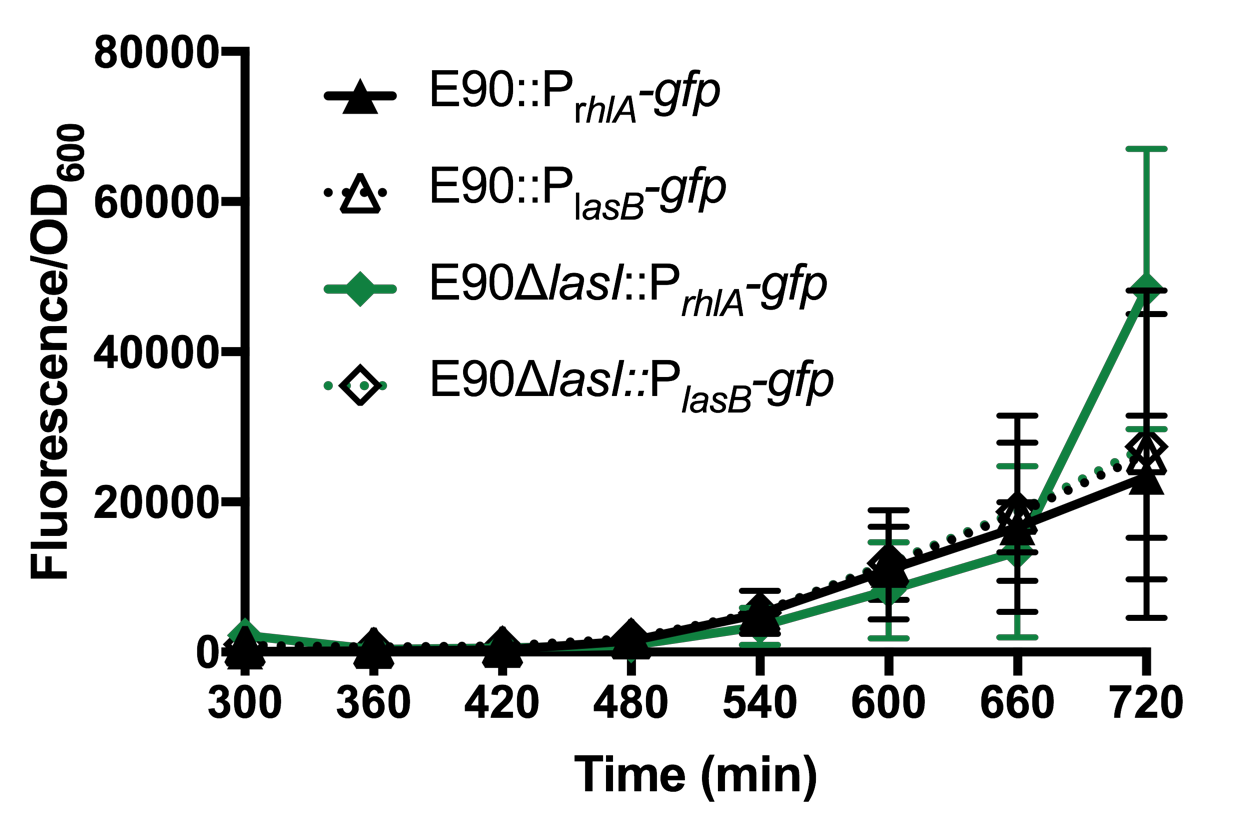


**Fig. S1. QS activity in E90 is not dependent on *lasI*.** Expression of *lasB* or *rhlA* in either E90 or E90∆*lasI*. Data from the first five hours are excluded because cell density measurements were below the limit of detection of the plate reader. Error bars represent the standard deviation for results of three independent experiments. In some cases, error bars are too small to be seen.
