# Supplementary material for: RhlR-Regulated Acyl-Homoserine Lactone Quorum Sensing in a Cystic Fibrosis Isolate of Pseudomonas aeruginosa
Source: mBio. 2020 Apr 7;11(2):e00532-20. doi: 10.1128/mBio.00532-20 (PMC7157775; doi:10.1128/mBio.00532-20)
Supplement: FIG S2 [file mBio.00532-20-sf002.docx]

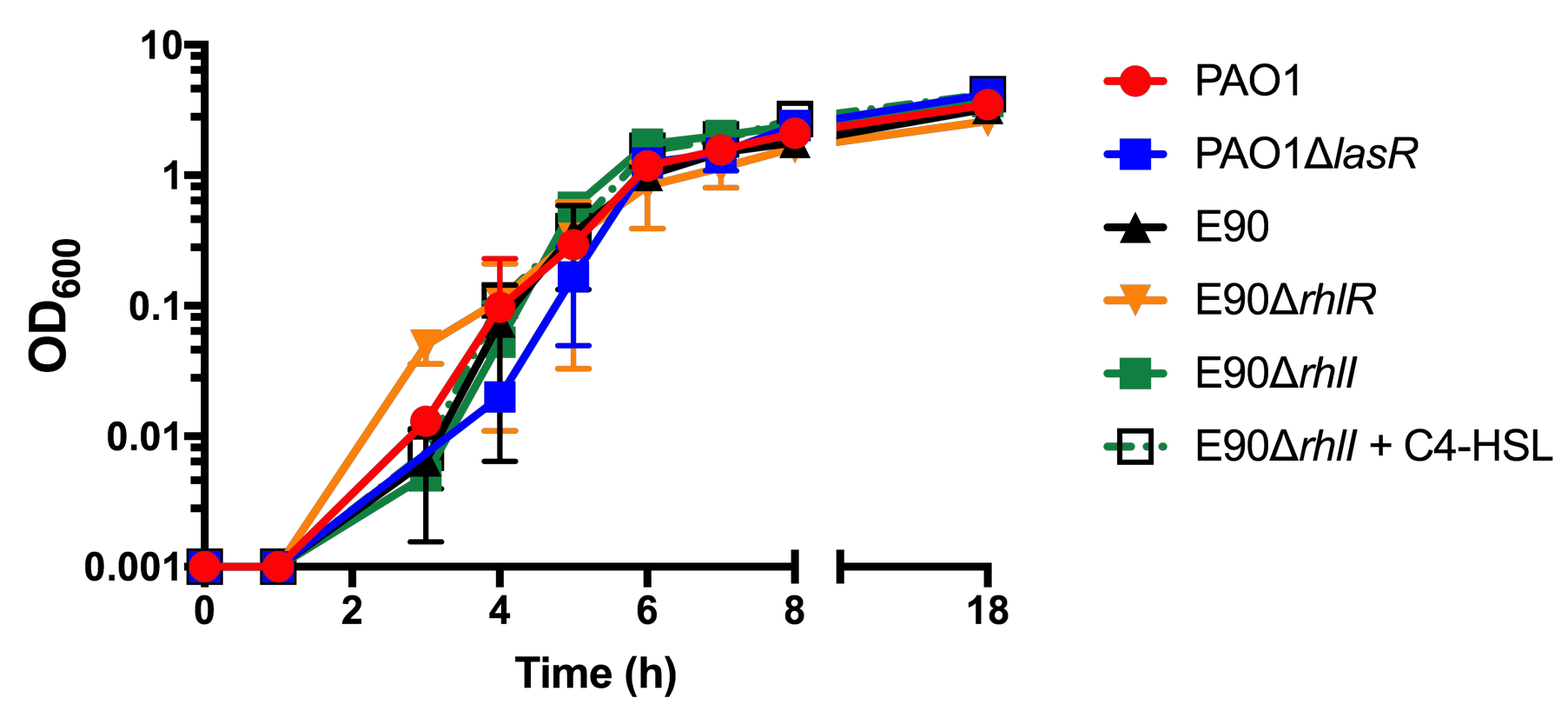


**Fig. S2. Growth curves of PAO1, E90, and PAO1 or E90-derived mutants in 3 mL of MOPS-buffered Luria Bertani broth.** Error bars represent the standard

deviation for results of three independent experiments. In some cases, error bars are

too small to be seen.
