# Supplementary material for: RhlR-Regulated Acyl-Homoserine Lactone Quorum Sensing in a Cystic Fibrosis Isolate of Pseudomonas aeruginosa
Source: mBio. 2020 Apr 7;11(2):e00532-20. doi: 10.1128/mBio.00532-20 (PMC7157775; doi:10.1128/mBio.00532-20)
Supplement: FIG S3 [file mBio.00532-20-sf003.docx]

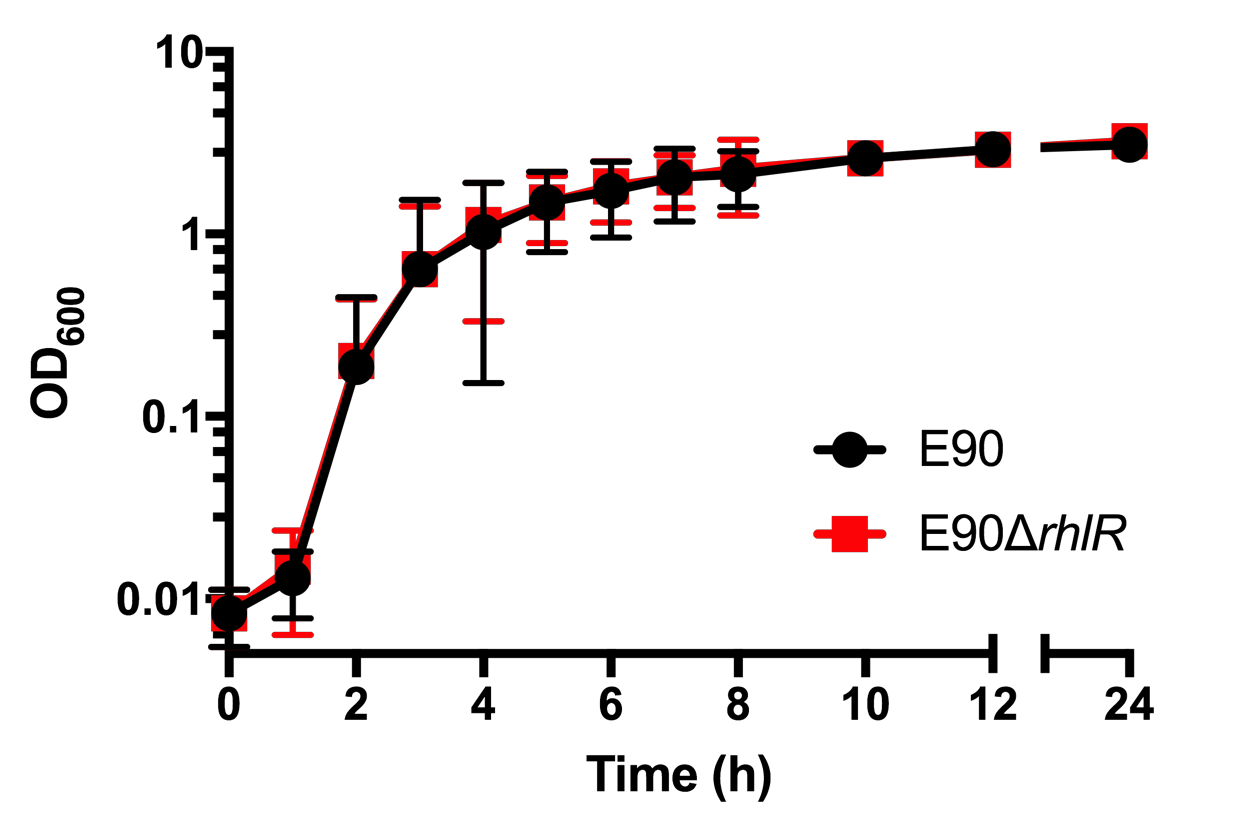


**Fig. S3. Growth curves of E90 and E90Δ*rhlR* in buffered Luria-Bertani Broth in 125-mL baffled flasks.** Means and standard deviation of biological replicates are shown (n=3). In some cases, error bars are too small to be seen.
